# Post-Mastectomy Flat Closure: A Mixed-Methods Analysis of Patient Outcomes and Perspectives

**DOI:** 10.1245/s10434-025-17288-8

**Published:** 2025-04-18

**Authors:** Daniel Soroudi, Nisha Parmeshwar, Aileen Gozali, Merisa Piper

**Affiliations:** 1 School of Medicine, University of California San Francisco, San Francisco, CA USA; 2 Division of Plastic and Reconstructive Surgery, Department of Surgery, University of California San Francisco, San Francisco, CA USA

**Keywords:** Post-mastectomy flat closure, Patient satisfaction, BREAST-Q survey, Complications and quality of life, Mixed-methods analysis

## Abstract

**Background:**

Understanding the psychosocial impact of mastectomy is crucial for guiding patient decisions on breast reconstruction. The BREAST-Q survey has been instrumental in assessing patient satisfaction. This study examines patient-reported outcomes and experiences with post-mastectomy flat closure, aiming to refine decision-making support for this option.

**Methods:**

A retrospective review analyzed flat closure outcomes post-mastectomy, examining patient demographics, surgical details, and complications. Following consent, patients completed the BREAST-Q survey, rating satisfaction and factors influencing their choice for flat closure. The study also included qualitative analysis from patient emails, employing an inductive, triangulated approach to extract themes and provide a multifaceted understanding of the post-surgical experience.

**Results:**

Overall, 252 patients underwent flat closure post-mastectomy. The overall postoperative complication rate was 17.5% (*n* = 44); 19.8% (*n* = 50) completed the BREAST-Q survey, reporting high satisfaction with their surgeons, moderate satisfaction with their breasts, and high physical well-being. Survey results illuminated reasons for choosing flat closure, such as lower complication rates and avoidance of foreign objects. Furthermore, qualitative feedback from 15 email correspondents revealed some limitations of the survey’s relevance and language, highlighting emotional impacts and a desire to improve future surveys.

**Conclusions:**

This study distills the flat closure experience post-mastectomy, highlighting its viability and alignment with patient preferences for fewer complications and quicker recovery. It reveals the BREAST-Q’s limitations in fully capturing patients’ nuanced experiences, underscoring the necessity for more tailored data collection methods. Future research must refine patient-reported outcome measures (PROMs) and ensure flat closure is a routinely discussed option in post-mastectomy care.

Breast reconstruction provides significant psychosocial benefits to patients after mastectomy.^1^ Understanding patient perceptions post-mastectomy is crucial for guiding decision making.^2,3^ Patient-reported outcome measures (PROMs) allow for evaluation of the patient experience. These methodological tools enable healthcare professionals to better understand patient perceptions and ultimately deliver high-quality, patient-centered care.^4,5^

The BREAST-Q survey, pivotal in gauging postoperative outcomes in breast surgery, was developed in 2009 through a rigorous process involving patient interviews and input from plastic surgeons and health professionals.^6,7^ It remains a relevant and widely used tool in patient-reported outcomes in breast cancer care, known for its comprehensive coverage of the physical, psychological, and quality of life (QoL) aspects experienced by patients.^8^

While implant-based breast reconstruction is the most common form of post-mastectomy reconstruction, in recent years it has become increasingly common for patients to choose ‘going flat’ after mastectomy.^9,10^ At our institution, all patients meet with a plastic surgeon to discuss all post-mastectomy reconstruction options, including implant-based reconstruction, autologous reconstruction, or flat closure. A prior study looking at patient perceptions after flat closure found faster recovery time and avoidance of foreign body placement as the two driving factors for choosing flat reconstruction;^11^ yet the lack of comprehensive research in this area precludes a clear understanding of decision-making processes in post-mastectomy flat closure patients. This gap in knowledge provides a compelling rationale for further investigation, warranting a detailed exploration of patient-reported outcomes among patients who choose to go flat.

By employing a mixed-methods approach, combining quantitative analysis and qualitative document analysis, this study explores clinical outcomes after post-mastectomy flat closure and evaluates patient satisfaction and QoL metrics in this cohort. Furthermore, this study aims to evaluate the applicability of the BREAST-Q survey for this patient population based on patient feedback. Through this comprehensive approach, the objective was to better understand patient-reported outcomes and experiences in the context of flat closure following mastectomy, ultimately with the goal of better counseling patients regarding this reconstruction option.

## Methods

### Study Design

A retrospective review was performed for patients who underwent post-mastectomy flat closure at our institution from January 2014 to June 2022. This study was approved by the University of California, San Francisco (UCSF) Institutional Review Board (IRB 19-29615).

Demographic variables were recorded, including age, race, body mass index (BMI), smoking status, cancer diagnosis, radiation or chemotherapy, and medical comorbidities. Psychosocial history documented included anxiety, depression, bipolar disorder, post-traumatic stress disorder (PTSD), suicidal ideations, and schizophrenia. Breast surgical history was recorded for any prior breast reduction, mastopexy, augmentation, lumpectomy, or mastectomy. Surgical characteristics included unilateral or bilateral surgery and simple, skin-sparing, or nipple-sparing mastectomy, as well as the specialty of the surgeon performing the flat closure. Operative notes were reviewed to identify specific flat closure techniques, such as de-epithelialization of mastectomy flaps, suspension to the pectoralis major muscle, and use of rotational skin flaps. Complications included seroma >20 cc requiring intervention, hematoma, nipple-areolar complex or skin flap necrosis, and surgical site infection (SSI) requiring oral and/or intravenous antibiotics. Adverse events not fitting these predefined categories were classified under ‘Other Complications’ to capture unanticipated or rare occurrences, ensuring a comprehensive assessment of complications observed during the study.

### BREAST-Q Survey

All identified patients who underwent post-mastectomy flat closure were emailed a ‘Survey Assessing Patient Satisfaction After Mastectomy’, which contained the study’s explanation and a consent form for signature. After obtaining informed, electronic, written consent, a link was securely provided for the BREAST-Q survey, with permission from Q-Portfolio. Three reminder emails were sent to patients who did not respond to the initial inquiry.

Through the BREAST-Q, education levels, indications for mastectomy, pre-surgery bra size, and reasons for choosing flat closure were recorded. Applicable breast reconstruction modules were included in the survey, which included two patient satisfaction domains (‘satisfaction with breasts’ and ‘satisfaction with surgeon’) and three QoL domains (‘psychosocial well-being’, ‘sexual well-being’, and ‘physical well-being’). A cumulative score from these five domains was then quantitatively translated into a 0–100 score per the BREAST-Q scoring tables. In addition to the standard domains, the survey incorporated three tailored queries: How happy are you with the outcome of your breast surgery?; How would you rate your overall health now?; and Why did you choose to go flat? Responses to these questions were recorded utilizing distinctive Likert scales, and subsequently compiled and reported for further analysis.

Each domain within the BREAST-Q survey was independently evaluated. The median scores, along with their interquartile ranges (IQRs), were calculated for further analysis. An independent t-test for equality of means was conducted to analyze differences between the participant and non-participant groups. Additionally, Mann–Whitney U tests were utilized to compare breast satisfaction scores by preoperative breast size, categorized as smaller (cup sizes A–B) or larger (cup sizes C and up), and by mastectomy laterality (unilateral vs. bilateral). Statistical analyses were executed utilizing IBM SPSS version 28.0 (IBM Corporation, Armonk, NY, USA).

### Qualitative Analysis

The qualitative portion of this study was centered on analyzing patient communications, specifically a set of 15 patient-initiated emails sent to the research investigator responsible for the study survey. Data analysis was initiated with a grounded theory informed approach, employing open coding of the transcript.^12^ Each phrase had the potential to generate novel codes, facilitating the emergence of meaningful insights directly from the data. These codes were subsequently organized into broader categories based on their shared content or meanings. This inductive approach allowed for the categories to be further grouped into overarching themes, or ‘parent codes’, to elucidate patterns pertinent to the research question. Each parent code, along with its associated codes, was then scrutinized and defined to clearly articulate its relevance and association with the research objectives.

To enhance validity and reliability, investigator triangulation was applied, where codes and themes were cross-verified among team members. The coding process was systematically recorded using memos and reflexive journaling. An audit trail was maintained to document the raw data, coding process, and theme development, thereby increasing the transparency and replicability of the research. Ethical guidelines were adhered to throughout the study, including obtaining necessary permissions, redacting identifiable information, and interpreting the data in a manner that respected the patients’ experiences and minimized bias.

## Results

### Retrospective Review

A total of 252 patients were identified who underwent flat closure after mastectomy, with a mean age of 61.4 ± 12.3 years at mastectomy. The mean time between mastectomy and the last plastic surgery follow-up was 832.7 ± 797.4 days. All participants were female, and the racial distribution was predominantly White (53.6%) and Asian (25.8%) (Table 1); 220 (87.3%) patients spoke English as their primary language.

**Table 1:** Patient demographics and medical history

	Patients (*N* = 252)
Age at mastectomy, years [mean ± SD]	61.4 ± 12.3
Race	
White	135 (53.6)
Asian	65 (25.8)
Other, declined, or unknown	35 (13.9)
Latinx	12 (4.8)
Pacific Islander	7 (2.8)
Black	5 (2.0)
BMI, kg/m^2^ [mean ± SD]	26.7 ± 6.1
Comorbidities	
Hypertension	82 (32.5)
Diabetes	30 (11.9)
Smoking status	
Never smoker	182 (72.2)
Former smoker	63 (25.0)
Current smoker	7 (2.8)
Psychosocial history	
Depression	41 (16.3)
Anxiety	34 (13.5)
Bipolar 1 or 2	3 (1.2)
Schizophrenia	1 (0.4)
PTSD	0 (0.0)
Suicidal ideations	0 (0.0)
Surgical history	
Partial mastectomy	61 (24.2)
Excisional biopsy	23 (9.1)
Mastectomy	13 (5.2)
Reconstruction	8 (3.2)
Reduction	5 (2.0)
Implant	3 (1.2)
Reduction	5 (2.0)
Mastopexy	1 (0.4)

Data are expressed as *n* (%) unless otherwise specified

*SD* Standard deviation, *BMI* Body mass index, *PTSD* Post-traumatic stress disorder

Overall, 157 (62.3%) patients underwent unilateral mastectomy, while 95 (37.7%) underwent bilateral procedures (Table 2). Among the unilateral flat closure group, 13 (8.3%) had a history of prior contralateral mastectomy and 10 (6.4%) underwent a concurrent contralateral procedure (eight reductions and two mastopexies). Five patients (3.2%) in the unilateral group and two patients (2.1%) in the bilateral group underwent delayed reconstruction, of which three were implant-based and four were autologous. External prostheses were prescribed more frequently for patients undergoing unilateral mastectomy (66 patients, 42.0%) than for those undergoing bilateral mastectomy (22 patients, 23.2%). Simple mastectomy was the most common surgical approach, performed on 84.1% of patients. 124 (49.2%) flat closures were performed by plastic surgeons and 128 (50.8%) by breast surgeons. Operative notes demonstrated variability in flat closure techniques, reflecting approaches tailored to individual patient needs and surgeon preferences. Commonly employed techniques included de-epithelialization of mastectomy flaps to optimize chest wall contour, suspension of flaps to the pectoralis major muscle to enhance chest wall shape, and the use of rotational skin flaps to augment soft tissue coverage, particularly in patients using external prostheses.

**Table 2:** Treatment details and postoperative complications

	Patients (*N* = 252)
Mastectomy state	
Unilateral	157 (62.3)
Bilateral	95 (37.7)
Type of mastectomy	
Simple mastectomy	212 (84.1)
Skin-sparing mastectomy	22 (8.7)
Total skin-sparing (nipple-sparing) mastectomy	18 (7.1)
Radiation treatment	
Yes	55 (21.8)
No	197 (78.2)
Chemotherapy	
Yes	50 (19.8)
No	202 (80.2)
Postoperative complications	44 (17.5)
Hematoma	19 (7.5)
Seroma	12 (4.8)
SSI requiring oral and/or intravenous antibiotics	5 (2.0)
Nipple or skin necrosis	1 (0.4)
Other	7 (2.8)

Data are expressed as *n* (%)

*SSI* Surgical site infection

Adjuvant therapies included radiation, administered to 21.8% of patients, and chemotherapy, administered to 19.8% of the cohort. The overall postoperative complications rate was 17.5% (*n* = 44), with specific complications including seroma (4.8%), hematoma (7.5%), nipple or skin necrosis (0.4%), and SSI requiring oral and/or intravenous antibiotics (2.0%). Other complications (2.8%) included SSI not requiring antibiotics (0.8%), postoperative anemia (0.8%), bloody nipple discharge (0.4%), neuroma (0.4%), and pulmonary embolism (0.4%).

### BREAST-Q Survey

Of 252 identified patients, 58 (23.0%) completed the consent form, with 50 (19.8%) fully completing the BREAST-Q survey. None of the BREAST-Q surveys were completed following delayed reconstruction. On average, the surveys were completed 4.6 ± 2.4 years after the mastectomy procedure. The mean age at mastectomy was noticeably higher in the non-participant group (62.6 ± 11.8 years) than in the participant group (56.2 ± 13.0 years) (*p* = 0.002). Specific characteristics of participants in the BREAST-Q survey, based on the self-reported data of the 50 patients, are reported in Table 3. The most common indication for mastectomy was invasive cancer, as reported by 86.0% of the patients. Carcinoma in situ accounted for 8.0% and prophylactic risk reduction accounted for 6.0%. The reasons for choosing flat closure after mastectomy varied among respondents, with the most common being the lower complication rate (76.0%) and shorter recovery time (70.0%) (Table 3). Furthermore, 70.0% of respondents cited avoidance of foreign objects in their body as a reason for flat closure. Other reasons included lower health risks (64.0%), body image independent of breasts (46.0%), avoidance of other body area procedures (40.0%), and social environment or personal knowledge influences (16.0%). Doctor’s advice or recommendation (14.0%), direct costs of the procedure (4.0%), indirect costs of the procedure (4.0%), and lack of option for reconstruction (8.0%) were less frequently cited reasons for choosing flat closure.

**Table 3:** Self-reported characteristics of questionnaire participants

	Participants (*N* = 50)
Education level	
Some college, trade, or university	2 (4.0)
Completed a college, trade, or university diploma	21 (42.0)
Some Master/Doctoral degree	3 (6.0)
Completed a Master/Doctoral degree	23 (46.0)
Other	1 (2.0)
Indication for mastectomy	
Invasive cancer	43 (86.0)
Carcinoma in situ	4 (8.0)
Prophylaxis	3 (6.0)
Bra size	
A	7 (14.0)
AA	3 (6.0)
B	15 (30.0)
D	6 (12.0)
C	15 (30.0)
DD (E)	1 (2.0)
DDD (F) or larger	3 (6.0)
Reason for flat closure	
Lower complication rate	38 (76.0)
Shorter recovery time	35 (70.0)
Avoidance of foreign objects	35 (70.0)
Lower health risks	32 (64.0)
Body image independent of breasts	23 (46.0)
Avoidance of other body area procedures	20 (40.0)
Influence of social environment or personal knowledge	8 (16.0)
Doctor’s advice or recommendation	7 (14.0)
Lack of option for reconstruction	4 (8.0)
Indirect costs of procedure	2 (4.0)
Direct costs of procedure	2 (4.0)

Data are expressed as *n* (%)

**Fig. 1:**
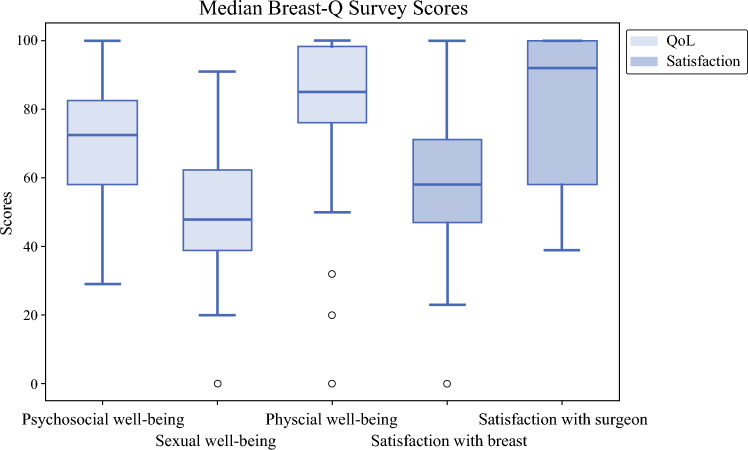
Median BREAST-Q Survey Scores

The two domains in the BREAST-Q were scored and analyzed (Fig. 1). Among the Patient Satisfaction domain, satisfaction with the surgeon showed the highest score with a median of 92 [IQR 58–100], while satisfaction with breasts scored lower at 58 [IQR 47–71]. Similarly, no significant difference was observed between the unilateral and bilateral mastectomy groups (58 [IQR 41.5–71] vs. 58 [IQR 48–73.8]; *p* = 0.834). In the QoL domain, physical well-being scored highest with a median of 85 [IQR 76–98]. Psychosocial well-being of the chest followed with a median of 72.5 [IQR 58–82.3], and sexual well-being had the lowest score with a median of 48 [IQR 39–62]. Regarding additional questions asked in the BREAST-Q survey, 66.0% of respondents reported being ‘extremely happy’, ‘very happy’, or ‘a little happy’ with their surgical outcomes, while 34.0% indicated that they were ‘extremely unhappy’, ‘very unhappy’, or ‘a little unhappy’ (Table 4). Furthermore, 86.0% of participants rated their postoperative health as ‘good’, ‘very good’ or ‘excellent’, 14.0% considered it ‘fair’, and notably, 0.0% rated their overall health as ‘poor’. Additionally, comparisons of breast satisfaction scores by preoperative breast size and mastectomy laterality revealed no significant differences. Patients with smaller breasts (cup size A–B) and larger breasts (cup size C and up) reported comparable satisfaction scores (58 [IQR 48–67.5] vs. 58 [IQR 44–71]; *p* = 0.911). Similarly, no significant difference was observed between the unilateral and bilateral mastectomy groups (58 [IQR 41.5–71] vs. 58 [IQR 48–73.8]; *p* = 0.834). Furthermore, there was no statistically significant difference in breast satisfaction scores between patients whose flat closures were performed by breast surgeons and plastic surgeons (64 [IQR 48–82] vs. 53 [IQR 46–64.5]; *p* = 0.358).

**Table 4:** Patient-reported outcomes

Overall happiness with outcome of surgery	Participants (*N* = 47)
Extremely unhappy	1 (2.1)
Very unhappy	7 (14.9)
A little unhappy	8 (17.0)
A little happy	7 (14.9)
Very happy	22 (46.8)
Extremely happy	2 (4.3%)
Overall rating of current overall health	Participants (*N* = 50)
Poor	0 (0.0)
Fair	7 (14.0)
Good	10 (20.0)
Very good	22 (44.0)
Excellent	11 (22.0)

Data are expressed as *n* (%)

### Qualitative Analysis

The quantitative data were supplemented by qualitative feedback from 15 respondents who engaged directly with the researcher via email, expressing various concerns and perspectives (Tables 5 and 6). These respondents expressed reservations about the relevance of the BREAST-Q survey to their specific situation. The qualitative document analysis identified four main categories emerging from the participant responses: *Study Participation, Reasons for Declining Participation, Negative Language and Emotional Impact of Survey, and Desire to Help Improve*. All four categories were grouped into one of two themes: *Respondent Intentions and Suggestions* or *Survey Experience and Feedback*. These categories illuminated a unique aspect of the participant experience with the survey and their engagement or lack thereof.

**Table 5:** Thematic analysis results— definitions

Category/code	Definitions
Study participation	Represents participants’ interactions with the research team, signifying their intent to participate or abstain
Interest in participation	Participant demonstrates an inclination to participate in the research study
Decline participation	Participant indicates a resolution to abstain from the research study
Unwanted communication	Participant communicates a preference to discontinue further correspondence
Desire to help improve	Participant displays interest in augmenting the research study’s progress through suggestions, professional insights, or other mechanisms
Specific suggestions for improvement	Highlights moments where participants offer concrete recommendations to enhance the study’s questionnaire or structure
Professional insight and feedback	Participant delivers feedback grounded on professional competence
Desire to share feedback about survey	Participant seeks a direct conversation or telephonic discussion
Reasons for declining participation	Reveals participants’ motivations for choosing to abstain from the research, such as eligibility, personal history, or other reasons
Eligibility concerns	Participant questions the eligibility prerequisites for participation in the research
No reconstruction	Participant declares a lack of relevant surgical history
Personal history	Participant divulges personal medical or familial narratives
Negative language and emotional impact of survey	Includes participants’ feedback related to the potential negative impacts of the survey’s language, including confusion or adverse emotional reactions
Confusion due to wording	Compiles participants’ responses where confusion or misunderstandings occur due to the survey’s language
Negative emotion from wording	Accumulates participants’ responses expressing discomfort or distress stemming from the survey’s terminology
Inaccurate representation	Gathers instances where participants feel the survey’s language does not mirror their experiences, causing feelings of misrepresentation
Feedback on terminology	Collects instances where participants offer specific feedback or suggestions regarding the survey’s language, indicating a desire for more precise or inclusive terminology
Dissatisfaction with survey scope	Assembles participants’ responses expressing dissatisfaction with the survey’s content depth or breadth, indicating the study does not sufficiently represent their experiences or situations

**Table 6:** Patient transcript quotes

Category/code	Quote	Patient ID
*Study participation*		
Interest in participation	“I would be very interested in participating in a study post-mastectomy… if I can be part of the [breast surgeon’s] surgery survey, I would be happy to participate” “I’m interested in participating in the research study of patient experience/satisfaction post-mastectomy”	1 8
Decline participation	“I have decided to not participate” “I’m sorry for the delay, my inbox is sometimes crazy. I have decided not to participate in the study”	13 14
Unwanted communication	“Please don’t send me this email again” “Did not have implant. Please stop messages”	15 10
*Desire to help improve*		
Specific suggestions for improvement	“UCSF should word the questions to be more specific about whether they are talking about the patient’s breast or chest” “My answers to the questions regarding the ‘breast cancer surgeon’ would be quite different for the oncology surgeon as compared to the plastic surgeon. Which do you mean? Would you like to add a separate question that splits them out?"	2 5
Professional insight and feedback	“As a researcher myself, I have some feedback on your survey. Especially related to the use of ‘go flat’ and the final question about ‘breast cancer surgeon.’ … If you ever want additional feedback about the general questionnaire/survey . . . I have many additional thoughts (especially as someone who was a PT … specializing in post-mastectomy treatment, a person whose Mom who has been a breast oncology nurse their whole life, and a survivor who also had a mastectomy in her 30s, and a queer person” “I worked for UCSF for many years. I believe this survey tool lacks the ability to capture the true experience of a mastectomy patient”	5 4
Desire to share feedback about survey	“I would like to discuss with you the proposed study. Please call me” “I’ve completed the survey but think it would be helpful to also discuss the issues with a researcher” “If you ever want additional feedback about the general questionnaire/survey . . . I have many additional thoughts (especially as someone who was a PT, a person whose Mom who has been a breast oncology nurse their whole life, and a survivor who also had a mastectomy in her 30s, and a queer person)”. “I have some specific feedback about the scope of the study, what was and wasn’t included in patient satisfaction questions, and I am requesting a meeting with the PI for the study to discuss my concerns"	7 4 5 6
*Reasons for declining participation*		
Eligibility concerns	“Please explain why I was recruited for the study and advise whether I qualify. P.S. And I did not have breast reconstruction. Both inclusion criteria are mentioned in the consent form” “I did not have reconstruction surgery. Do you still want me to participate?”	8 9
No reconstruction	“And I did not have breast reconstruction” “I did not have reconstruction surgery” “Did not have implant” “I did not have any reconstructive surgery, so I don’t think I am who you are looking for” “Just FYI, I opted out of reconstruction, so this is not relevant to my experience”	8 9 10 11 12
Personal history	“I do not have a history of breast cancer”	8
*Negative language and emotional impact of survey*		
Confusion due to wording	“I completed the survey. It was confusing because it asked me to rate my surgeon. I had Dr. Ewing as my surgeon, and Dr. Piper as my Plastic Surgeon. Since this survey is from Dr. Piper, I answered the questions re my experience of Dr. Piper” “My answers to the questions regarding the ‘breast cancer surgeon’ would be quite different for the oncology surgeon as compared to the plastic surgeon. Which do you mean?”	3 5
Negative emotion from wording	“Just on a side note for your future surveys. That part of the survey triggered me a little bit. UCSF should word the questions to be more specific about whether they are talking about the patient’s breast or chest” “I find the use of ‘go flat’ to be dismissive of the complications and nature of the decision not to have reconstruction, which you seem to understand given the follow-up questions. This made me want to stop the survey right there. Did you test this out on populations who’ve experienced this surgery and check that language?”	2 5
Inaccurate representation	“There is no place to indicate if you are not sexually active. I made a note of that in the OTHER box, I used to answer a different question”	3
Feedback on terminology	“UCSF should word the questions to be more specific about whether they are talking about the patient’s breast or chest” “As a researcher myself, I have some feedback on your survey. Especially related to the use of ‘go flat’ and the final question about ‘breast cancer surgeon’ …. My answers to the questions regarding the ‘breast cancer surgeon’ would be quite different for the oncology surgeon as compared to the plastic surgeon. Which do you mean? Would you like to add a separate question that splits them out?”	2 5
Dissatisfaction with survey scope	“There is no room for nuance or personal anecdotes in the survey and, as such, it feels very impersonal and incomplete” “I have some specific feedback about the scope of the study, what was and wasn’t included in patient satisfaction questions, and I am requesting a meeting with the PI for the study to discuss my concerns” “I worked for UCSF for many years. I believe this survey tool lacks the ability to capture the true experience of a mastectomy patient” “There is no place to indicate if you are not sexually active. I made a note of that in the OTHER box, I used to answer a different question”	4 6 4 3

*UCSF* University of California San Francisco, *PI* Principal Investigator, *PT* Physical Therapist, *FYI* for your information

*Study Participation*: Responses in this area ranged widely from enthusiastic participation to outright decline, often rooted in perceived irrelevance or unwanted communication. One participant displayed interest by stating, “*I would be very interested in participating in a study post-mastectomy’*, whereas another exhibited resistance by requesting, “*Please don’t send me this email again*”.

*Reasons for Declining Participation*: Misunderstandings about the survey’s relevance and comprehension of the questions largely accounted for participants’ declining involvement. For instance, some participants expressed concerns about their eligibility. Notably, five patients contacted us believing the survey did not apply to them due to not having undergone reconstruction. One patient’s email stated, “*Please explain why I was recruited for the study and advise whether I qualify; P.S. and I did not have breast reconstruction*”.

*Negative Language and Emotional Impact of Survey*: Some participants noted confusion, negative emotions, and feelings of misrepresentation arising from the survey’s wording and language use. As one participant described, “*I find the use of ‘go flat’ to be dismissive of the complications and nature of the decision not to have reconstruction*”. Another participant noted that the wording was triggering*.* “*Just on a side note for your future surveys. That part of the survey triggered me a little bit. [You] should word the questions to be more specific about whether they are talking about the patient’s breast or chest*”. Additionally, four patients were dissatisfied with the scope of the survey, as it did not capture key details regarding their experience. One patient wrote, “*There is no room for nuance or personal anecdotes in the survey and, as such, it feels very impersonal and incomplete*”*.*

*Desire to Help Improve*: Despite the challenges, many participants expressed a willingness to contribute to improving the survey. They offered suggestions, and professional insights, and even expressed a desire for more direct modes of communication. A participant with professional research experience wrote, “*As a researcher myself, I have some feedback on your survey, especially related to the use of ‘go flat’ and the final question about ‘breast cancer surgeon’* ”. Another patient requested a direct meeting with the principal investigator of this study to offer specific feedback. “*I have some specific feedback about the scope of the study, what was and wasn’t included in patient satisfaction questions, and I am requesting a meeting with the PI for the study to discuss my concerns*”*.*

## Discussion

This single-institution experience with post-mastectomy flat closure outcomes, as well as the results of the BREAST-Q survey in this patient population, provides further insights regarding patient perceptions of the survey. The mixed-methods approach employed in this study encompasses both quantitative analysis and qualitative document analysis, allowing for a multifaceted exploration of patient experiences after post-mastectomy flat closure. A patient’s decision-making process is fundamentally influenced by a range of elements such as potential complications, recovery time, body image, and individual personal circumstances, and this mixed-methods approach evaluates each of these from various viewpoints.

In this patient cohort, the overall complication rate was 17.5%, lower than the rates of 22% and 37% found for subpectoral and prepectoral implant patients, respectively, in a 2023 study conducted at the same institution.^13^ The rate of SSI requiring oral and/or intravenous antibiotics was 2.0% for the flat closure method, compared with 6% and 8% for the subpectoral and prepectoral patients respectively.^13^ Furthermore, flat reconstruction completely avoids the potential of donor site complications inherently present with autologous reconstruction.^14^ While direct comparisons are challenging due to the paucity of data specifically examining flat closure as a distinct procedure, existing literature provides benchmarks for related procedures. Murphy et al. reported an 18.6% complication rate for mastectomy alone, encompassing 474 patients across three studies, suggesting a similar risk profile to our flat closure cohort.^15^ This implies that the specific techniques employed in flat closure, at least in our institution, do not appear to substantially elevate complication risks beyond those associated with standard mastectomy. Conversely, Bennett et al. observed a significantly higher complication rate of 32.9% in a large cohort of 2343 patients undergoing post-mastectomy reconstruction.^16^This contrast highlights the potential for increased complications when more extensive procedures, such as those involved in reconstruction, are performed. Lower complications may explain why an increasing number of patients are showing a preference for the flat closure method, aligning with this study’s finding of ‘lower complication rate’ being the most common reason for flat closure. The growing ‘going flat’ movement may reflect a shift towards greater patient autonomy in making post-mastectomy decisions, rather than it being a consequence of limited reconstructive choices. Yet, literature has cited that up to 22% of women wishing to undergo flat reconstruction experienced ‘flat denial’ by either being left with excess chest tissue or not being offered flat closure as an option.^11,17,18^ Simply offering flat closure as an option is not sufficient. The variability in satisfaction scores observed in our study, with 34.0% of patients reporting some level of unhappiness with their surgical outcome, underscores that flat closure may not be the optimal choice for every patient. Greater satisfaction has been associated with receiving adequate information about surgical options, highlighting the critical need for individualized counseling and shared decision making.^11^These discussions should consider each patient’s unique values, preferences, and expectations. Factors such as pre-existing body image concerns, expectations, and coping mechanisms may also influence postoperative satisfaction and should be explored during preoperative consultations.^19^ Patients can thus be more empowered to make choices that align with their preferences, even if those preferences evolve over time, as seen in a small subset of our patients (2.8%) who later underwent delayed reconstruction.

The quantitative data from the electronic health records and the BREAST-Q survey provided valuable insights into patient preferences and experiences. The decision to choose flat closure after mastectomy was influenced by factors such as lower complication rates, shorter recovery time, avoidance of foreign body placement, and individual personal circumstances. While physical well-being and satisfaction with surgeons scored highest in the QoL and Patient Satisfaction domains, respectively, our analysis also revealed concerningly low scores for sexual well-being and satisfaction with breasts. Interestingly, satisfaction with breasts did not significantly differ based on preoperative breast size or laterality of mastectomy. This suggests that factors beyond the physical dimensions of the breast, such as individual perceptions of aesthetic outcomes, the use of external prostheses, and, potentially, experiences of ‘flat denial’, may play a more significant role in shaping overall satisfaction with the decision to go flat. For instance, the higher utilization of external prostheses among unilateral mastectomy patients (42.0% vs. 23.2%) could indicate an attempt to mitigate aesthetic and functional concerns related to asymmetry, potentially influencing their satisfaction levels. However, the low scores in sexual well-being and breast satisfaction warrant further investigation, particularly when compared with the meta-analysis by Shauly et al. that reported higher satisfaction in these domains among patients who underwent implant-based or autologous reconstruction.^20^ While the limitations of the BREAST-Q, which was originally designed for patients undergoing breast reconstruction, likely contribute, other factors are probably at play. Some patients may struggle with adapting to a body without breasts, even if they chose flat closure. This could be exacerbated by societal pressures surrounding femininity and the prevalent notion that breasts are integral to a woman’s identity. One patient’s comment that the term ‘go flat’ felt “dismissive of the complications and nature of the decision not to have reconstruction” highlights the emotional complexity surrounding this choice. Despite these concerns, most patients expressed satisfaction with certain aspects of their flat closure experience, supporting the viability of flat closure as an option for mastectomy patients. Our study found that a majority of patients who underwent flat closure expressed satisfaction with their surgical outcomes, with most rating their postoperative health as good, very good, or excellent. This aligns with the findings of Baker et al., who reported a mean satisfaction score of 3.72 out of 5 among women active in online ‘Going Flat’ communities, and that 74.1% of their respondents indicated that mastectomy alone was their first choice.^11^ While our study did not utilize the same satisfaction scale, the generally positive self-reported health ratings and the overall satisfaction expressed by our participants suggest a comparable level of contentment with flat closure.

This study’s response rate of 20% was markedly lower than the 72% response rate reported in BREAST-Q’s field-testing study.^21^ This low response rate may be attributed to the survey’s perceived irrelevance to patients who opted for flat closure, as several respondents expressed concerns about its applicability to their specific experiences. Given that the BREAST-Q survey was initially developed with a focus on more traditional forms of breast reconstruction, its domains may not fully capture the unique considerations and outcomes most relevant to those choosing flat closure. To improve the tool’s utility in this population, we recommend including items specific to flat closure, such as satisfaction with chest contour, the absence of breasts, and the use of external prostheses. Including more open-ended questions would also allow patients to share their experiences in their own words, providing richer qualitative data. Focus groups and semi-structured interviews with patients who have undergone flat closure would be an excellent starting point for developing these new items. Furthermore, some study respondents expressed concerns about the survey’s applicability, language use, and its potential to trigger negative emotions. This prompts a re-evaluation of the survey’s appropriateness for diverse reconstructive populations and underscores the importance of clear and considerate communication in survey development. The feedback and suggestions provided by patients demonstrate a strong investment in the refinement and improvement of research tools. Thus, this study not only addresses the specific needs of flat closure patients but also contributes to the broader understanding of patient-reported outcomes, which are challenging to obtain.

This study expands research on patient-reported outcomes in breast cancer care. While previous studies have looked at BREAST-Q surveys regarding other forms of breast reconstruction, this study addresses the experiences of patients who opted for flat reconstruction.^22–24^ However, this study is not without its limitations. While the response rate itself provides valuable insights, it also represents a limitation, as the findings may not fully capture the diverse range of patients’ experiences. Furthermore, while the survey was sent in English, 12.7% of patients did not speak English as their primary language, which partly explains the low response rate. This highlights the need for future research to prioritize translating surveys and consent forms into multiple languages to ensure inclusivity and improve response rates from diverse populations. The retrospective design and varying intervals between the procedure and survey may introduce recall bias, as satisfaction and perceptions can evolve over time. Additionally, the absence of baseline measures or normative data limits the ability to make direct comparisons or assess changes in satisfaction. Efforts were made to contextualize findings using similar studies; however, this limitation remains important to consider. The qualitative feedback from a limited number of participants, which was derived from patient-initiated correspondence, provides valuable insights but may overrepresent strong positive or negative experiences. As such, these findings may not fully capture the breadth of perspectives on flat closure post-mastectomy.

Flat closure is associated with high psychosocial well-being scores and lower overall complication rates, reinforcing its viability as a regular option for patients undergoing mastectomy. The development of more inclusive PROMs, through focus groups and semi-structured interviews, is crucial for accurately capturing and addressing the experiences of flat closure patients. This will ultimately contribute to improved patient care and satisfaction.

## Conclusions

This mixed-methods study provides a comprehensive insight into the experiences and outcomes of patients who opted for flat closure after mastectomy. The study reaffirms that flat closure is a viable option with generally lower complication rates compared with other forms of reconstruction, aligning well with patient-centric reasons for this choice, such as fewer complications and shorter recovery time. This study adds needed quantitative and qualitative data on the often-overlooked flat closure population. However, the study also exposes gaps in patient satisfaction and the limitations of existing survey tools such as the BREAST-Q in capturing the nuanced experiences of this specific population, which is needed as flat closure continues to gain traction. Furthermore, clinicians must engage in shared decision making, ensuring that flat closure is presented as a routinely discussed option alongside reconstruction, empowering patients to make informed choices that align with their individual values and preferences.
